# High-Resolution Cortical Dipole Imaging Using Spatial Inverse Filter Based on Filtering Property

**DOI:** 10.1155/2016/8404565

**Published:** 2016-08-29

**Authors:** Junichi Hori, Shintaro Takasawa

**Affiliations:** ^1^Graduate School of Science and Technology, Niigata University, Niigata 950-2181, Japan; ^2^Terumo Corporation, Tokyo, Japan

## Abstract

Cortical dipole imaging has been developed to visualize brain electrical activity in high spatial resolution. It is necessary to solve an inverse problem to estimate the cortical dipole distribution from the scalp potentials. In the present study, the accuracy of cortical dipole imaging was improved by focusing on filtering property of the spatial inverse filter. We proposed an inverse filter that optimizes filtering property using a sigmoid function. The ability of the proposed method was compared with the traditional inverse techniques, such as Tikhonov regularization, truncated singular value decomposition (TSVD), and truncated total least squares (TTLS), in a computer simulation. The proposed method was applied to human experimental data of visual evoked potentials. As a result, the estimation accuracy was improved and the localized dipole distribution was obtained with less noise.

## 1. Introduction

The spatial resolution of electroencephalogram (EEG) data is limited because of the small number of scalp surface electrodes used and the low conductivity of the skull. Therefore, it was difficult to specify brain electrical activity directly from the potential distribution measured on the scalp surface. Cortical dipole imaging that estimates the equivalent dipole source distribution on a virtual layer within a brain from the scalp potential has been proposed to solve this problem [1, 2]. According to cortical dipole imaging, brain electrical activity is represented by the equivalent dipole distribution without being restricted in the number and the direction of the signal sources.

The cortical dipole distribution is estimated from the scalp potentials by solving an inverse problem of the transfer matrix from the dipole layer to the scalp surface based on a head model. The solution of the inverse problem is influenced not only by the measurement noise but also by the error in the transfer matrix. The measurement noise originates in the measurement environment, caused by factors such as the variance of the electrode impedance, the environmental noise, and artifacts caused by eye blinks or body movements. On the other hand, the transfer matrix error originates in the distortion of the head model design such as errors of an electrode displacement, individual differences in head shape, and nonuniform electrical conductivity. Therefore, it is important to reduce the influence from both the measurement noise and the transfer matrix error for the EEG inverse solution of cortical dipole imaging.

Several spatial inverse filters have been proposed to reduce the influence of the measurement noise. Tikhonov regularization [3] and truncated singular value decomposition (TSVD) [4] were applied to truncate the noisy components. Use of a parametric projection filter incorporated with the statistical information on the noise has also been proposed [5, 6]. Moreover, the transfer matrix error was taken into consideration in the truncated total least squares (TTLS) method [7]. In this method, after scaling the covariance of the transfer matrix error to equal that of the measurement noise, the solution is estimated by minimizing the influence from both the measurement noise and the transfer matrix error. TTLS has been applied to a bioluminescence topography inverse problem [8] and an ECG inverse problem [9]. We applied TTLS to the inverse problem of cortical dipole imaging [10]. TTLS provided better results compared with the traditional inverse methods when the transfer matrix was included in the forward problem.

In the present study, we paid attention to filtering property when solving the cortical inverse problem in order to improve the accuracy of cortical dipole imaging. Filtering property presents the amplitude characteristics by changing the singular value when singular value decomposition (SVD) is applied to the inverse solution. According to filtering property, the terms easily influenced by noise are reduced while the terms with less noise are passed. We estimated optimum filtering property using the least squares method (LS) in the simulation of several signal source configurations. An inverse filter model was constructed by approximating the filtering property with a sigmoid function. The proposed method was compared with traditional inverse techniques such as Tikhonov regularization, TSVD, and TTLS in computer simulation [11]. Based on the simulation results, our method was applied to human EEG data of visual evoked potential (VEP) [12]. The results indicated that the proposed method can provide better performance compared with traditional inverse techniques. In the present study, the restorative ability and applicability of the proposed method are defined by changing the signal and noise configurations and by applying these to several sets of experimental data. Concretely, the optimum parameter for the sigmoid function-based inverse filter is investigated by changing the depth of the signal source and the noise level.

## 2. Methods

### 2.1. Cortical Dipole Imaging

**Figure 1:**
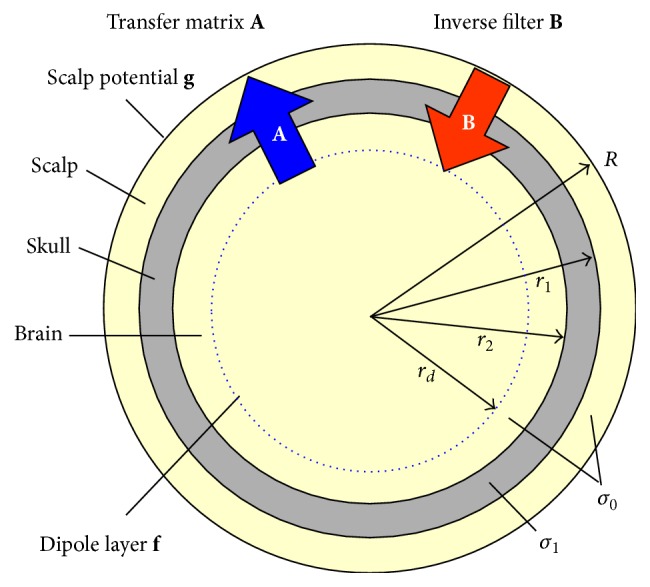
3 sphere inhomogeneous volume-conductor head model.

Cortical dipole imaging is one of the high-resolution EEG mapping techniques. A volume-conductor head model is used to estimate the cortical dipole distribution from measured scalp potentials. The head model is approximated by an inhomogeneous set of three concentric spheres that represent the scalp, the skull, and the brain, as shown in Figure 1 [2]. The radius of the scalp is set to 1 and the radii of the skull and the cortex are set to 94% and 87% of the scalp radius, respectively. The conductivity of the skull was set to *σ*_1_ = 0.0125 and the conductivity of the cortex and the scalp was *σ*_0_ = 1.0. A dipole layer was established inside of the cortex with arbitrary radius, *r*_*d*_. A total of 1280 equivalent radial dipoles were uniformly arranged on the dipole layer to represent the dipole signal sources in a brain. Cortical dipole imaging has an advantage that there is no restriction on the number and direction of dipole sources.

The observation of the scalp potential **g** is modeled using the transfer matrix **A** from the dipole layer to the scalp surface as follows:

(1) $$\begin{array}{c} g=\left(\;A+E\;\right)f+n, \end{array}$$

where **f** is the dipole distribution, **E** is the transfer matrix error, and **n** is the measurement noise. The transfer matrix **A** is determined from the geometry of the head model, the electrical conductivity involved, and the electrode and equivalent dipole source arrangements. The inverse problem should be solved to estimate the dipole distribution $\hat{f}$ from the measured scalp potential **g**:

(2) $$\begin{array}{c} \hat{f}=Bg, \end{array}$$

where **B** is the inverse filter. As the method to construct the inverse filter, Tikhonov regularization [3] and TSVD [4] were proposed to reduce the influence of measurement noise. In addition, the TTLS method that reduces both the measurement noise and the transfer matrix error was investigated [7].

### 2.2. Inverse Techniques

The *m* × *n* transfer matrix is decomposed by

(3) $$\begin{array}{c} A={U\Sigma V}^{T}, \end{array}$$

where **U** and **V** are *m*th- and *n*th-orthogonal matrices and Σ is the singular value matrix:

(4) Σ=zij∈Rm×n,zij=σi,for  i=j,0,for  i≠j,σ1≥⋯≥σr>0,σr+1=⋯=σn=0.

*σ*_*i*_  (*i* = 1,…, *n*) represents singular values. **u**_*i*_ and **v**_*i*_ are left and right singular vectors, respectively. The parameter *r* is the number of nonzero singular values.

In general, the inverse problem is solved by LS. The solution is described by

(5) $$\begin{array}{c} {\hat{f}}_{\text{LS}}=\overset{r}{\underset{i=1}{\sum }}\frac{u_{i}^{T}g}{\sigma_{i}}v_{i}. \end{array}$$

According to this method, the noise and error involved in the scalp potential** g** are enhanced by the terms of small singular values. Tikhonov regularization and TSVD that estimate the solutions while suppressing the influence from the measurement noise overcome this problem. Tikhonov regularization is given by

(6) $$\begin{array}{c} {\hat{f}}_{\text{TIKH}}={\left(\;A^{T}A+\gamma I\;\right)}^{-1}A^{T}g=\overset{r}{\underset{i=1}{\sum }}\frac{\sigma_{i}^{2}}{\sigma_{i}^{2}+\gamma^{2}}\frac{u_{i}^{T}g}{\sigma_{i}}v_{i}, \end{array}$$

where *γ*  (*γ* > 0) is the regularization parameter [3]. TSVD is given by

(7) $$\begin{array}{c} {\hat{f}}_{\text{TSVD}}=\overset{k}{\underset{i=1}{\sum }}\frac{u_{i}^{T}g}{\sigma_{i}}v_{i}, \end{array}$$

where *k* is the truncation parameter [4]. When the error is involved in the transfer function, the estimated solutions have bias in these methods. To reduce the influence of the transfer function error, TTLS was introduced [7]. The SVD for the augmented matrix (**A**, **g**) is given by

(8) A,g=U− Σ− V−T,U−=u−1,…,u−m,V−=v−1,…,v−n+1,U− U−T=Im,V− V−T=In+1,Σ−=z−ij∈Rm×n+1,z−ij=σ−i,for  i=j,0,for  i≠j,σ−1≥⋯≥σ−r>0,σ−r+1=⋯=σ−n=0.

When *k* is a truncated parameter, the solution of TTLS is given by

(9) $$\begin{array}{c} {\hat{f}}_{\text{TTLS}}=-{\overset{-}{V}}_{12}{\overset{-}{V}}_{12}^{+}=-\frac{{\overset{-}{V}}_{12}{\overset{-}{V}}_{22}^{T}}{{\left\|{\overset{-}{V}}_{22}\right\|}_{2}^{2}}, \end{array}$$

where

(10) $$\begin{array}{c} \overset{-}{V}=\left(\begin{array}{cc} {\overset{-}{V}}_{11} & {\overset{-}{V}}_{12}\\ {\overset{-}{V}}_{21} & {\overset{-}{V}}_{22} \end{array}\right),\;{\overset{-}{V}}_{11}\in R^{n\times k},\;\;{\overset{-}{V}}_{22}\in R^{1\times \left(n+1-k\right)}. \end{array}$$

**V** ^+^ is the Moore-Penrose pseudoinverse of **V**. The notation ‖  ‖_2_ denotes the Euclidian norm. In TTLS, the measurement noise and the transfer function error are combined by the augmented matrix. After decomposing the augmented matrix, the singular values that enhance the measurement noise and the transfer matrix error are truncated.

### 2.3. Filtering Property

These inverse solutions of Tikhonov regularization, TSVD, and TTLS are commonly expressed as

(11) $$\begin{array}{c} \hat{f}=\overset{r}{\underset{i=1}{\sum }}p_{i}\frac{u_{i}^{T}g}{\sigma_{i}}v_{i}, \end{array}$$

where *p*_*i*_ is a filter factor. The filter factor {*p*_*i*_}  (*i* = 1,…, *r*) is called a filter property [7, 11]. The filter factors *p*_*i*_ of Tikhonov regularization, TSVD, and TTLS are derived by

(12) pi,TIKH=σi2σi2+γ2,pi,TSVD=1,for  i=1,…,k,0,for  i=k+1,…,r,pi,TTLS=∑j=1kv−n+1,j2V−2222σi2σ−j2−σi2.

**Figure 2:**
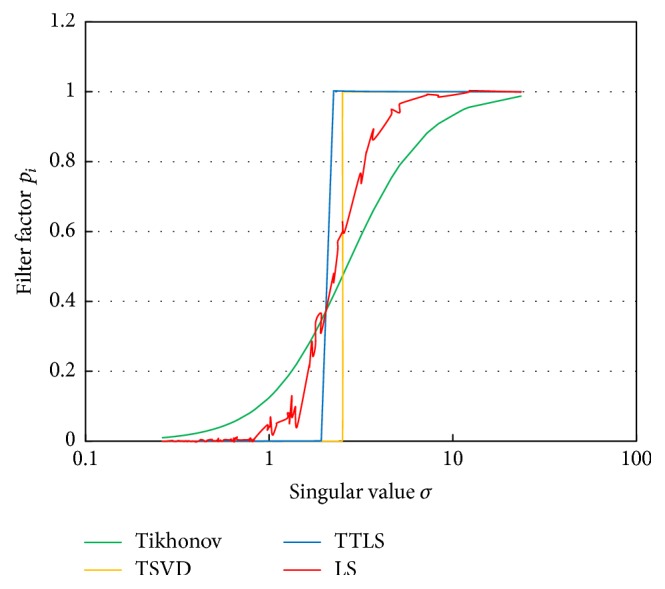
Filter factors of various inverse techniques against the singular value.

Figure 2 shows examples of the filter factors of Tikhonov regularization, TSVD, and TTLS against the singular values. The eccentricity of the signal sources was set to 0.6. Ten percent measurement noise and 10% transfer matrix error were added to the scalp potential and the transfer matrix, respectively. The radius of the dipole layer was set to 0.85. The singular value on the horizontal axis is displayed using a logarithmic scale. The terms for large singular values were passed while the terms for small singular values were attenuated in all filter factors. While the property of Tikhonov regularization was gradual, the properties of TSVD and TTLS were steep.

### 2.4. Optimal Filtering Property Using Sigmoid Function

When the actual dipole distribution is known, it is possible to find optimal filtering property by LS. The estimated optimal filtering property is shown in Figure 2. Filter factors were calculated using 100 kinds of signal source arrangement to adapt to various signal configurations. The estimated optimal filter property was intermediate between the property of Tikhonov regularization and the property of TSVD, as shown in Figure 2.

**Figure 3:**
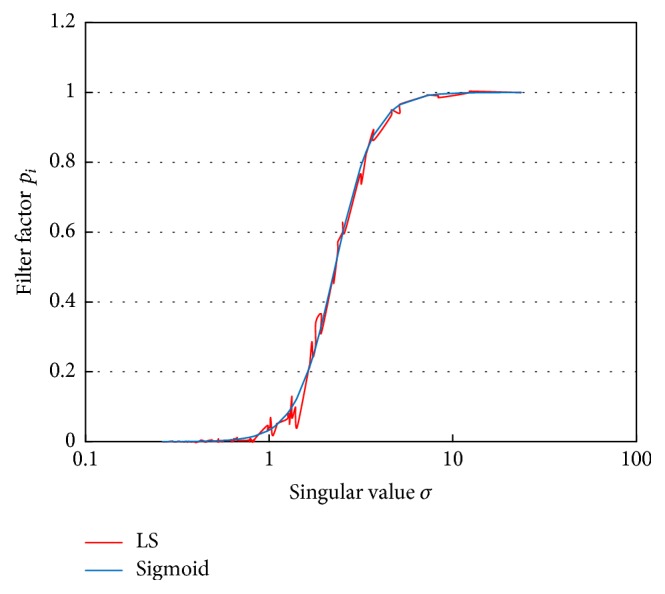
Optimal filter factors approximated by sigmoid function.

The filter factors have fluctuation because they were calculated using sampled signal sources. We approximated the filter property by a sigmoid function:

(13) $$\begin{array}{c} p_{i}=\frac{1}{1+e^{-a\left(\log\sigma_{i}-\gamma \right)}}=\frac{1}{1+\sigma_{i}^{-a}e^{a\gamma }}, \end{array}$$

where *a* is a gain parameter and *γ* is a shift parameter. The approximated result of optimal filter factors by (13) is also shown in Figure 3. The optimal filtering property was changed according to the level of measurement noise, the transfer matrix error, and the depth of the signal sources. However, it is possible to approximate the filter factor by (13) with appropriate two parameters, *a* and *γ*.

**Figure 4:**
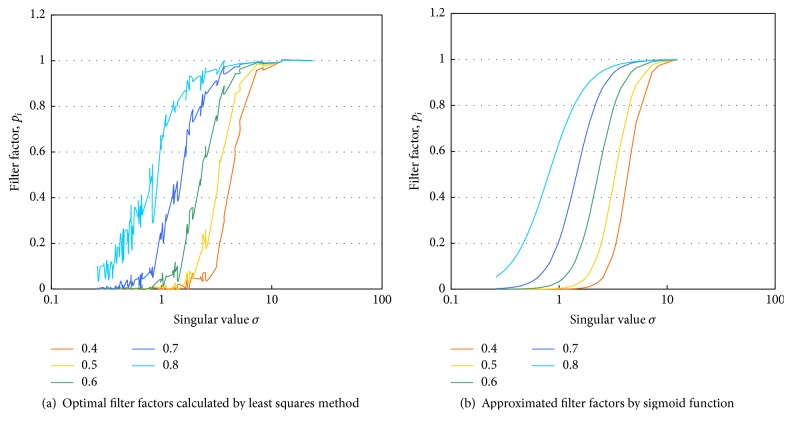
Optimal filter factors by changing the depth of signal sources.

The aspect of the dipole distribution changes according to the depth of the signal source against the fixed dipole layer. The dipole distribution spreads so that the signal source is located in a deep position. The optimal filter when changing the depth of the signal source within the range 0.4–0.8 was estimated using the LS. The noise level was set to 0.1. The calculated optimal filter factors are plotted in Figure 4(a). The deeper the signal source was, the more singular values were suppressed by the filter property. It was confirmed that larger singular values were suppressed when the noise level was large. The sigmoid function in (13) was fitted to the obtained optimal filter factors as shown in Figure 4(b). The filter factors were well approximated with appropriate parameters.

**Figure 5:**
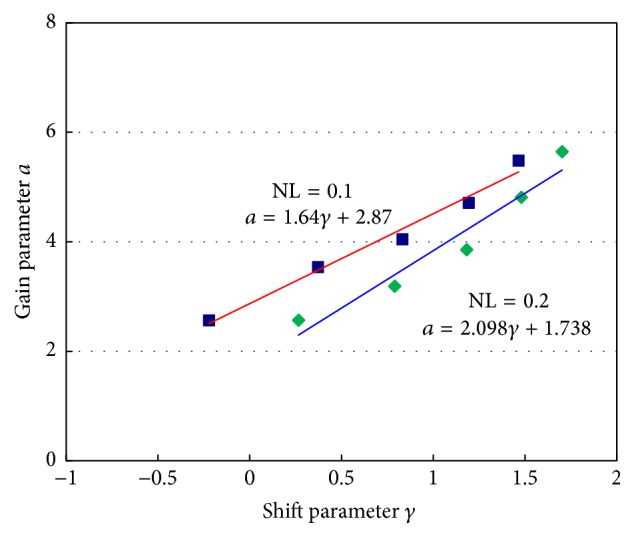
Relationship between shift parameter *γ* and gain parameter *a* in (9).

In (13), it is necessary to estimate 2 parameters. There are calculation costs associated with this method, compared with traditional methods consisting of only 1 parameter. Thus, we investigated the relationship between 2 parameters. If one parameter is estimated, the other can be decided using the derived relationship equation. The relationship between 2 optimal parameters was repeatedly investigated by changing the depth of signal sources with a constant noise level. The relationship between the regularization parameter and the shift parameter in the sigmoid function is plotted in Figure 5. The relationships between 2 parameters for noise levels of 0.1 and 0.2 were approximated by linear equations. As a result, the filter factors can be estimated using only shift parameter *γ*.

This parameter relationship changed according to the noise level. In order to use the sigmoid function to determine the filter factors in actual EEG data, the parameter relationship should be decided using the information on noise level. However, the signal and noise components are intermingled in the observed EEG data. In such cases, independent component analysis (ICA) was applied to the EEG data to separate in the signal and noise components. ICA extracts independent sources from the observed signal based on statistical independence of the original signal. In a previous study, the noise component was precisely estimated from the subtraction of the separated signal component from the observed EEG data [13]. That is, the components without a signal component were assumed to be noise. The noise level was calculated by ‖**n**‖_2_/‖**g**‖_2_.

### 2.5. Parameter Estimation

In actual application, the shift parameter *γ* in the sigmoid function has to be determined as the same as the regularization parameter *γ* in Tikhonov regularization and the truncated parameter *k* in TSVD and TTLS [14–20]. If the actual dipole distribution is known, the parameter can be determined by minimizing the relative error between actual and estimated dipole distribution. However, the actual dipole distribution is unknown in practical situations. For such cases, the L-curve method was proposed to estimate the regularization parameter [14, 15]. In the L-curve method, the optimal regularization parameter is decided by minimizing both the estimated solution norm ${\left\|\hat{f}\right\|}_{2}$ and the residual norm ${\left\|A\hat{f}-g\right\|}_{2}$. The optimal parameter corresponds to the corner of the L-curve. As an estimation method for the corner of the L-curve, a curvature method that searches for the point of maximum curvature was proposed [16]. A minimal product method that estimates a minimum of the area ${\left\|A\hat{f}-g\right\|}_{2}\cdot {\left\|\hat{f}\right\|}_{2}$ was also proposed [17].

## 3. Results

### 3.1. Simulations

Computer simulations were performed to evaluate the ability of the proposed inverse filter. A total of 128 electrodes were arranged uniformly on the scalp surface. The dipole layer with 1280 equivalent dipole sources was established with a depth of 0.85 inside of the brain. The numbers of electrodes and dipoles were set to be great enough to accomplish high spatial resolution, based on previous studies [2, 5]. Two radial signal sources with eccentricity of 0.6 were arranged with arbitrary position. Gaussian white noise was added as the measurement noise and the transfer matrix error.

**Figure 6:**
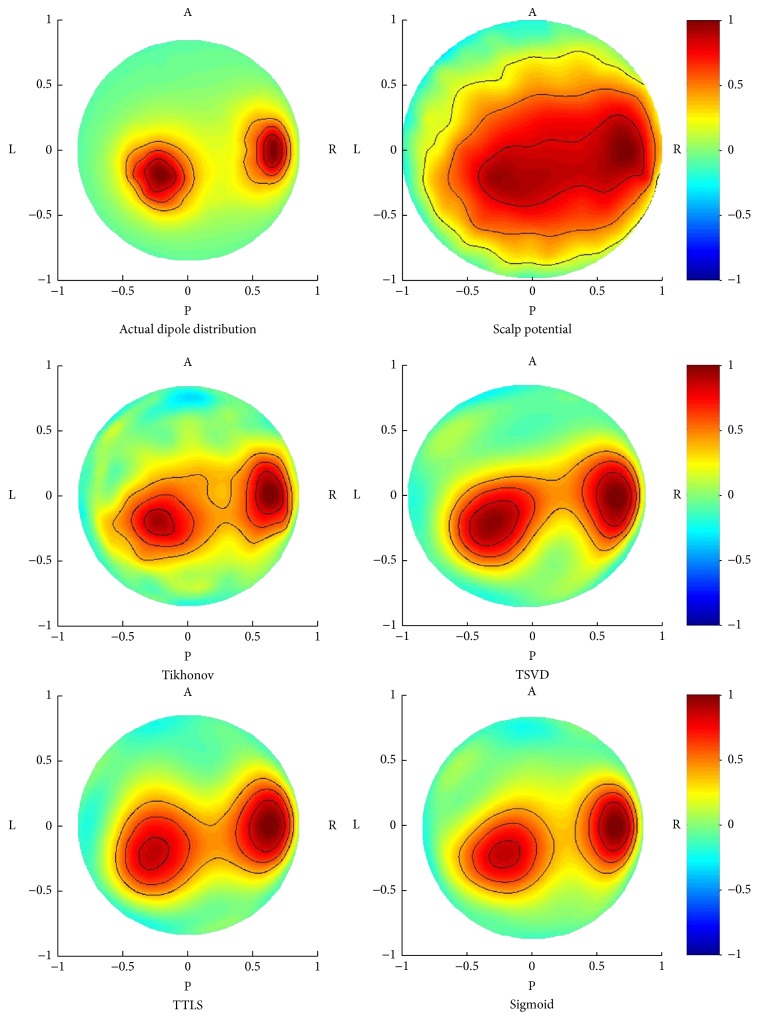
Simulation results of dipole distributions estimated by Tikhonov regularization, TSVD, TTLS, and sigmoid function. R: right; L: left; A: anterior; P: posterior.

First, we compared the inverse estimations of Tikhonov regularization, TSVD, TTLS, and the sigmoid function.  Figure 6 shows the estimated results of dipole distributions for 2 radial signal sources with a depth of 0.6. The noise level and the error level were both set to 0.1.  Figure 6 displays the top view of dipole distributions with normalized amplitude. Two signal sources could be observed in the actual dipole distribution while the distribution of the scalp potential was spread over whole parietal region. The dipole distributions were estimated using Tikhonov regularization, TSVD, TTLS, and the sigmoid function. To avoid the influence of parameter estimation error, the regularization parameters were determined by minimizing the relative error when the actual dipole solution is supposed to be known. The result of Tikhonov regularization was influenced by noise. The localization was accomplished by the sigmoid function compared with TSVD and TTLS. The performance of TSVD was almost the same as that of TTLS.

**Figure 7:**
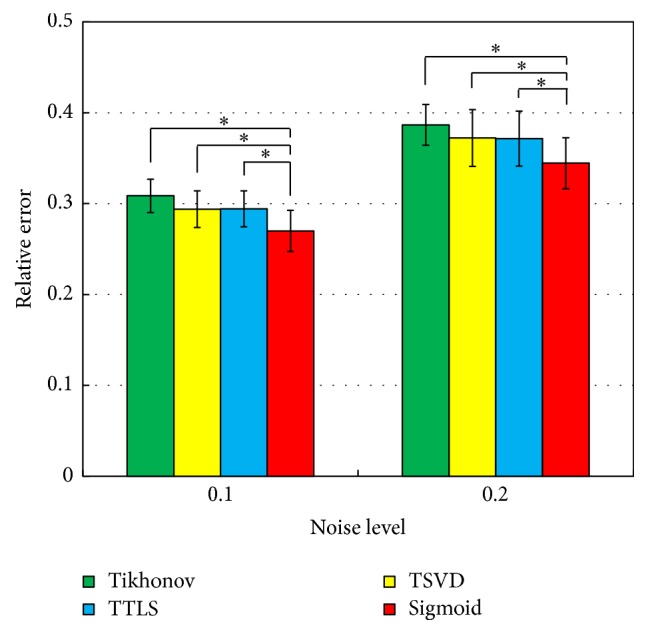
Relative errors of dipole distributions estimated by Tikhonov regularization, TSVD, TTLS, and sigmoid function (*N* = 10, *∗* < 0.05).

**Figure 8:**
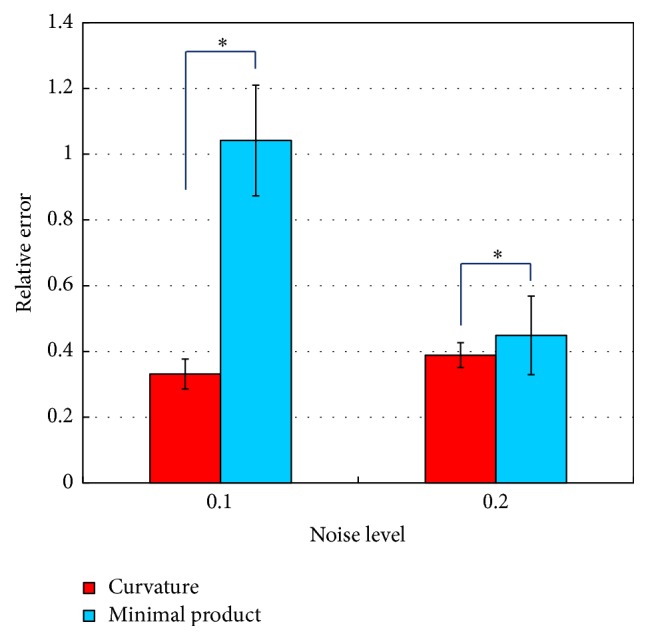
Comparison of parameter estimation methods (*N* = 10, *∗* < 0.05).

Figure 7 shows the averaged relative error between actual and estimated dipole distributions with noise levels of 0.1 and 0.2. The error level was set to 0.1. The graphs show the average and standard deviation over 10 patterns of signal source arrangements. Dunnett's multiple comparison tests were applied to the data. The relative error of the sigmoid function is significantly smaller than that of the other methods. The performance of parameter estimation methods was examined in computer simulations. The curvature method and the minimal product method were compared by means of the relative error between actual and estimated dipole distributions.  Figure 8 shows the relative errors with the noise levels of 0.1 and 0.2. The average and the standard deviation were obtained over 10 trials with various signal configurations. The relative error of the curvature method was significantly smaller than that of the minimal product method as a result of paired *t*-test. It was confirmed that the curvature method is more suitable for parameter estimation of sigmoid function than the minimal product method.

### 3.2. Application to VEP

Based on the simulation results, the proposed method was applied to human experimental data. The EEG data were measured from healthy subjects after obtaining informed consent according to the University of Illinois Ethical Review Board regulation. Ninety-four scalp electrodes arranged according to the expanded international 10-20 system were used for the EEG recording. The VEP to pattern reversal in the right half of the visual field was measured with intervals of 0.5 s. The VEP signals were averaged over 400 reversals. The sampling frequency was 1 kHz. The dipole layer was arranged with a radius of 0.85 to represent the visual-related signal sources. From the simulation result, the regularization parameter of the sigmoid function was estimated by means of the curvature method. Referring to a previous study, the curvature method was applied to Tikhonov regularization and the minimal product method was applied to TSVD and TTLS as the parameter estimation methods [10].

**Figure 9:**
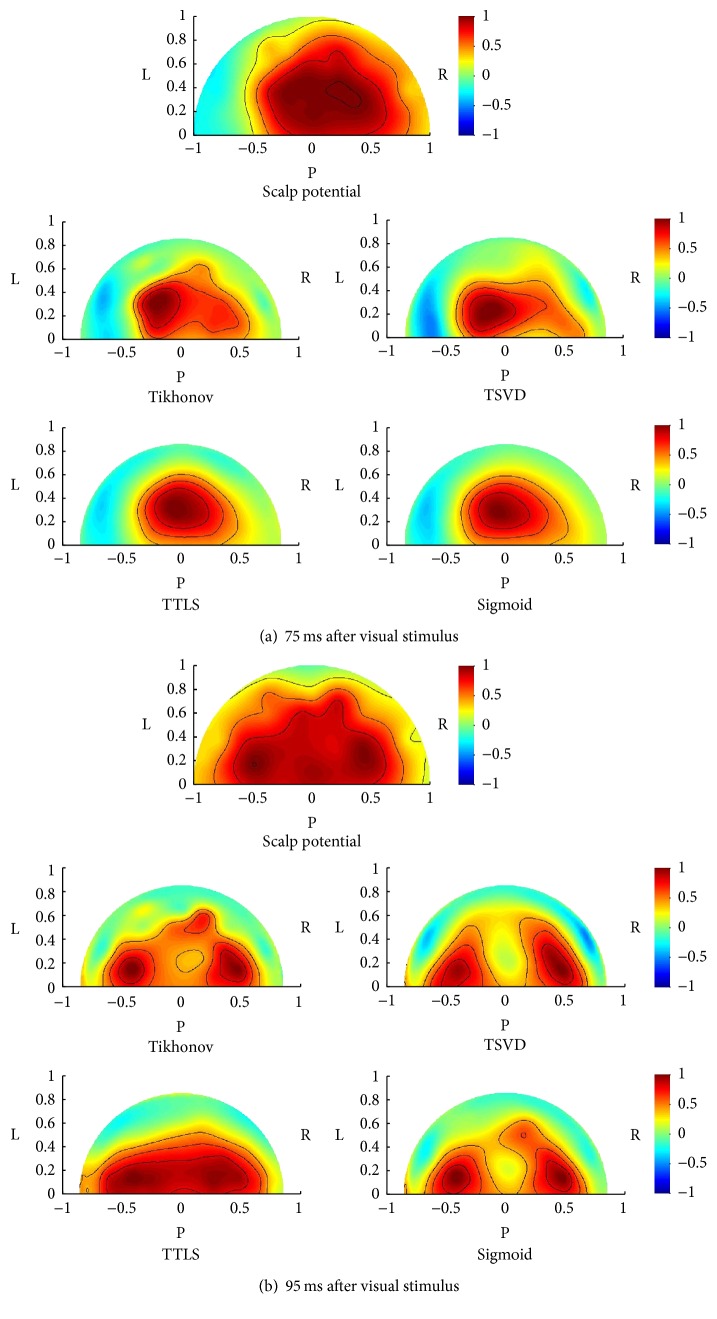
Estimated results of dipole distributions for VEP. R: right; L: left; P: posterior.

Positive potential was observed at about 100 ms after visual stimulus (P100). Cortical dipole imaging was applied at the positive peak (75 ms after the stimulus), followed by the propagation process (95 ms after the stimulus).  Figure 9 shows the scalp potential and the dipole distributions estimated by Tikhonov regularization, TSVD, TTLS, and the proposed sigmoid function at 75 ms and 95 ms after stimulus (the results at other time intervals are omitted for want of space). The normalized maps were displayed when viewing from the posterior. The positive potential was distributed over the whole occipital region in the scalp potential map at 75 ms after stimulus. The signal was localized at the primary visual field in dipole distributions estimated by every method. The influence of noise was observed in the results of Tikhonov regularization and TSVD. The signal was more localized when TTLS and the sigmoid function were used. The positive potential was also distributed over the whole occipital region in the scalp potential map at 95 ms after stimulus. Two signal spots were visible in dipole distributions. From the viewpoint of signal separation, TSVD and the sigmoid function provided better results than Tikhonov regularization and TTLS.

## 4. Discussion

As shown in (13), the optimal filtering property was approximated using a sigmoid function with the singular value as a variable parameter. In the inverse problem of cortical dipole imaging, the number of singular values decayed exponentially as the singular values became large. Thus, the sigmoid function was fitted to the optimal filtering property based on the logarithm of a singular value.

The deeper the signal sources, the broader the dipole distribution. Broad mapping can be approximated with small number of singular values compared with sharp mapping. As a result, the larger singular values were depressed when the signal sources were located in deep positions. Moreover, when the noise level is large, the noise component must be suppressed in order to obtain high-fidelity dipole distribution. We confirmed that the larger singular values were depressed in noisy conditions.

Whenever the noise level and the depth of signal sources were changed, it was possible to fit the sigmoid function to the optimal filter factors estimated using LS. The cortical dipole distribution using the sigmoid function was more localized with less noise compared with the traditional methods. When the noise level increased, the relative error of every method increased. However, the relative error of the sigmoid function was smallest among the 4 inverse filters investigated. From these results, it was considered that highly precise estimation for cortical dipole imaging could be achieved by optimizing the filter factors.

In general, small singular values emphasize the noise included in the scalp potentials. The terms with small singular values were suppressed by filtering properties as shown in Figure 1. In addition, as explained in Section 2.4, the filtering property of the sigmoid function was intermediate between Tikhonov regularization and TSVD. This filter attenuates the terms with small singular values to reduce the noise while it passes the terms of large singular values to reconstruct the signal. The sigmoid function performs the signal reconstruction and noise reduction in a well-balanced manner.

The filter property based on the sigmoid function takes into consideration only measurement noise, while TTLS considers both the measurement noise and the transfer matrix error. The present simulation, considering the measurement noise and the transfer matrix error, suggested that the optimum filter property that considered the measurement noise properly was effective to obtain better inverse solutions. It is expected that the restorative ability would be improved by combining the sigmoid function-based inverse filter with TTLS.

As the method for estimating the regularization parameter of the sigmoid function, the curvature method was better than the minimal product method. In the previous study, it was reported that the curvature method was used for continuous values such as the regularization parameter of Tikhonov regularization while the minimal product was used for discrete values such as the truncated parameter of TSVD and TTLS [10]. The shift parameter *γ* of the sigmoid function in (13) is a continuous value. Thus, the curvature method is suitable for the parameter estimation of the sigmoid function.

In the VEP experiment shown in Figure 9, the signal was localized at occipital region of cortical dipole distribution at 75 ms after visual stimulus. The activated area corresponds to the primary visual cortex and the results coincided with established physiological knowledge [21]. Both TTLS and the sigmoid function can localize the signal while suppressing the noise. The dipole distribution at 95 ms after visual stimulus showed two signals separated from one signal at 75 ms after visual stimulus. The visual signal caused at calcarine sulcus in the occipital region propagates through ventral and dorsal pathways [21]. It was possible to represent the process of signal propagation through ventral pathway using TSVD and the sigmoid function. In conclusion, regarding these two results, the sigmoid function was widely applicable in various situations and would be effective for human experimental data. It is difficult to evaluate the experimental performance quantitatively because the actual signal source is unknown. The experimental results of cortical dipole imaging have been evaluated visually in previous studies [10, 12, 13, 19]. The cortical imaging techniques may be evaluated by comparing with electrocorticogram invasively [22].

The proposed inverse filter supposed that the noise is uniformly distributed over the scalp surface. Actually, the noise is nonuniform because of the variation of the electrode impedance and physiological properties such as eye blink artifacts or body movements. In such cases, the parametric projection filter [5, 6] and parametric Wiener filter [23] can be effectively applied under nonuniform noise conditions. It is expected that cortical dipole imaging is improved by combining the sigmoid function-based filtering property and parametric projection or Wiener filter.

## 5. Conclusion

The spatial inverse filter was investigated based on filtering property aiming at high-resolution cortical dipole imaging. It was confirmed that the optimum filter factor depends on the noise level and the depth of signal sources. These results suggested that the filtering property can be designed by considering the signal and noise configuration. Moreover, the proposed method is of wide application for several types of experimental data. In computer simulations and human experiments using VEP, filtering property using the sigmoid function provided more localized dipole distribution with less noise compared with Tikhonov regularization, TSVD, and TTLS. For parameter estimation, the curvature method was suitable for the sigmoid function-based inverse technique. The proposed method will contribute to the visualization of cortical electrical activity in high resolution. We are planning to design filtering property using statistical information on noise distribution. Moreover, we would apply more realistic head models in the near future.

## References

[1] Sidman R. D., Ford M. R., Ramsey G., Schlichting C. (1990). Age-related features of the resting and P300 auditory evoked responses using the dipole localization method and cortical imaging technique. *Journal of Neuroscience Methods*.

[2] Wang Y., He B. (1998). A computer simulation study of cortical imaging from scalp potentials. *IEEE Transactions on Biomedical Engineering*.

[3] Tikhonov A. N., Arsenin V. Y. (1977). *Solutions of Ill-Posed Problems*.

[4] Hansen P. C. (1987). The truncated SVD as a method for regularization. *BIT Numerical Mathematics*.

[5] Hori J., He B. (2001). Equivalent dipole source imaging of brain electric activity by means of parametric projection filter. *Annals of Biomedical Engineering*.

[6] Hori J., Aiba M., He B. (2004). Spatio-temporal cortical source imaging of brain electrical activity by means of time-varying parametric projection filter. *IEEE Transactions on Biomedical Engineering*.

[7] Fierro R. D., Golub G. H., Hansen P. C., O'Leary D. P. (1997). Regularization by truncated total least squares. *SIAM Journal on Scientific Computing*.

[8] He X., Liang J., Qu X., Huang H., Hou Y., Tian J. (2010). Truncated total least squares method with a practical truncation parameter choice scheme for bioluminescence tomography inverse problem. *International Journal of Biomedical Imaging*.

[9] Shou G., Xia L., Jiang M., Wei Q., Liu F., Crozier S. (2008). Truncated total least squares: a new regularization method for the solution of ECG inverse problems. *IEEE Transactions on Biomedical Engineering*.

[10] Hori J., Takeuchi K. Cortical dipole imaging using truncated total least squares considering transfer matrix error.

[11] Takasawa S., Hori J. (2015). Improvement of cortical dipole imaging based on filtering property approximated by sigmoid function. *IEICE Technical Reports*.

[12] Takasawa S., Hori J. (2014). Improvement of cortical dipole imaging based on filtering property. *IEICE Technical Report*.

[13] Hori J., Watanabe Y. (2011). Cortical dipole imaging for multiple signal sources considering time-varying non-uniform noise. *IEEJ Transactions on Electronics, Information and Systems*.

[14] Subramaniyam N. P., Väisänen O. R. M., Wendel K. E., Malmivuo J. A. V. (2010). Cortical potential imaging using L-curve and GCV method to choose the regularisation parameter. *Nonlinear Biomedical Physics*.

[15] Hansen P. C., O'Leary D. P. (1993). The use of the L-curve in the regularization of discrete ill-posed problems. *SIAM Journal on Scientific Computing*.

[16] Hosoda Y., Kitagawa T. (1992). Optimum regularization for ill-posed problems by means of L-curve. *The Japan Society for Industrial and Applied Mathematics*.

[17] Lian J., He B. (2001). A minimal product method and its application to cortical imaging. *Brain Topography*.

[18] Castellanos J. L., Gómez S., Guerra V. (2002). The triangle method for finding the corner of the L-curve. *Applied Numerical Mathematics*.

[19] Rodriguez G., Theis D. (2005). An algorithm for estimating the optimal regularization parameter by the *L*-curve. *Rendiconti di Matematica, Serie VII*.

[20] Hansen P. C., Jensen T. K., Rodriguez G. (2007). An adaptive pruning algorithm for the discrete L-curve criterion. *Journal of Computational and Applied Mathematics*.

[21] Eyesenck M. W., Keane M. T. (2010). *Cognitive Psychology: A Student's Handbook*.

[22] He B., Zhang X., Lian J., Sasaki H., Wu D., Towle V. L. (2002). Boundary element method-based cortical potential imaging of somatosensory evoked potentials using subjects' magnetic resonance images. *NeuroImage*.

[23] Hori J., Miwa T., Ohshima T., He B. (2007). Cortical dipole imaging of movement-related potentials by means of parametric inverse filters incorporating with signal and noise covariance. *Methods of Information in Medicine*.

